# Validation of the Arabic language version of the Audio Processor Satisfaction Questionnaire (APSQ) for hearing implant users

**DOI:** 10.1371/journal.pone.0303301

**Published:** 2024-06-10

**Authors:** Abdulaziz Alasmi, Shaza Saleh, Ilona Anderson, Yassin Abdelsamad, Abdulrahman Hagr

**Affiliations:** 1 King Abdullah Ear Specialist Center (KAESC), King Abdulaziz University Hospital, King Saud University Medical City, Riyadh, Saudi Arabia; 2 Clinical Research Department, MED-EL GmbH, Innsbruck, Austria; 3 Research Department, MED-EL GmbH, Riyadh, Saudi Arabia; Hannover Medical School: Medizinische Hochschule Hannover, GERMANY

## Abstract

**Objective:**

Audio processors (AP) are the external components of hearing implants. User satisfaction with the performance and comfort of their AP is a critical factor in ensuring daily use, which leads to improved hearing outcomes. The aim of this study was to construct and validate an Arabic language translation of the APSQ for use among Arabic-speaking clinicians and patients.

**Design:**

The original APSQ was translated into the Arabic language using cross-cultural adaptation measures. The final questionnaire was administered to CI users in electronic form.

**Study sample:**

117 CI users (64 female) participated. A total of 179 ears were assessed.

**Results:**

High levels of satisfaction with audio processors were observed among CI users. Item and scale analyses indicate that this version of the APSQ measure a homogeneous and valid construct.

**Conclusion:**

The Arabic version of the APSQ captures user satisfaction with hearing implant audio processors.

## 1. Introduction

Implanted hearing devices encompass a wide array of technologies, including implants of the cochlea, middle ear and auditory brainstem, as well as bone conduction devices. Hearing implants are well-established interventions that provide numerous benefits, including improvements in sound perception, sound localization, speech understanding and communication, and overall quality of life [1–4].

The benefits from the use of these devices can be assessed using both objective and subjective measures. Objective measures use psychophysical test procedures to assess hearing outcomes, while subjective measures employ questionnaires and other self-reported metrics. While objective measures can yield more direct and quantitative data, the aspects of hearing which they measure can be rather narrow, such as the degree of speech perception in noise or the acuity of sound localization. In contrast, subjective measures can capture hearing outcomes in situations which are difficult to replicate in a formal testing environment. Several questionnaires have been devised to assess subjective hearing outcomes. These include the Speech, Spatial and Qualities of Hearing Scale (SSQ) [5], the Hearing Implant Sound Quality Index (HISQUI19) [6], and the Glasgow Benefit Inventory (GBI) [7].

An important component of hearing benefit is user satisfaction. A key aspect of user satisfaction lies with the audio processor (AP). As an externally worn device, the AP can have an impact on the user’s comfort and social life, and the user must assume responsibility for some aspects of its maintenance, such as cleaning and battery replacement.

Measures of AP satisfaction have been developed for specific models [8–10]. More recently, a model-agnostic questionnaire has been developed: the audio processor satisfaction questionnaire (APSQ) [11]. This tool measures user satisfaction using fifteen items grouped into three subscales (comfort, social life, and usability).

The APSQ has been administered to users of cochlear implants, middle ear implants, bone conduction devices, and electric-acoustic stimulation systems [11–17]. The APSQ was originally developed in the German language and has also been used in English. It can be used to complement objective measures of hearing outcomes, as well as other subjective measures of benefit such as quality of life, communication, functioning in everyday situations, and social life satisfaction [12–18].

Arabic is spoken by 274 million people—the sixth most spoken language worldwide. Despite this, many audiological tools are not available to be administered in this language. Here we attempt to remedy this in part by constructing and validating an Arabic language translation of the APSQ. We employed cross-cultural adaptation measures to translate the questionnaire items. We administered the APSQ to a large cohort of Arabic-speaking cochlear implant users including both children and adults. It is our hope that our work will contribute to the proliferation of this tool within the Arabic language area, as well as to encourage other translation efforts in this field.

## 2. Methods

### 2.1 Subjects

Subjects to be included in the study were required to have at least 4 months’ experience with their current audio processor and use Arabic as their first language. In total, 117 CI users (64 female) were included in this study between February and May 2022. The mean age at the time of study was 18 years, ranging from 1.8 to 60 years. 36 were unilateral CI users, 62 were bilateral users, and 19 were bimodal users. A total of 179 ears were included (94 from female). Four of these were excluded from some analyses due to insufficient data to calculate total scores, and four others were excluded entirely due to insufficient data to calculate sub-scores. Daily audio processor usage is shown in Table 1. For 68.2% (n = 122) of the ears, the audio processor was used for more than 12 hours per day.

**Table 1:** Daily device usage times recorded per ear.

Usage time (h)	Number	Percent
0–3	6	3.4
3–6	4	2.2
6–9	19	10.6
9–12	24	13.4
>12	122	68.2
Not recorded	4	2.2
Total	179	100

### 2.2. Translation of the APSQ into the Arabic language

**Fig 1:**
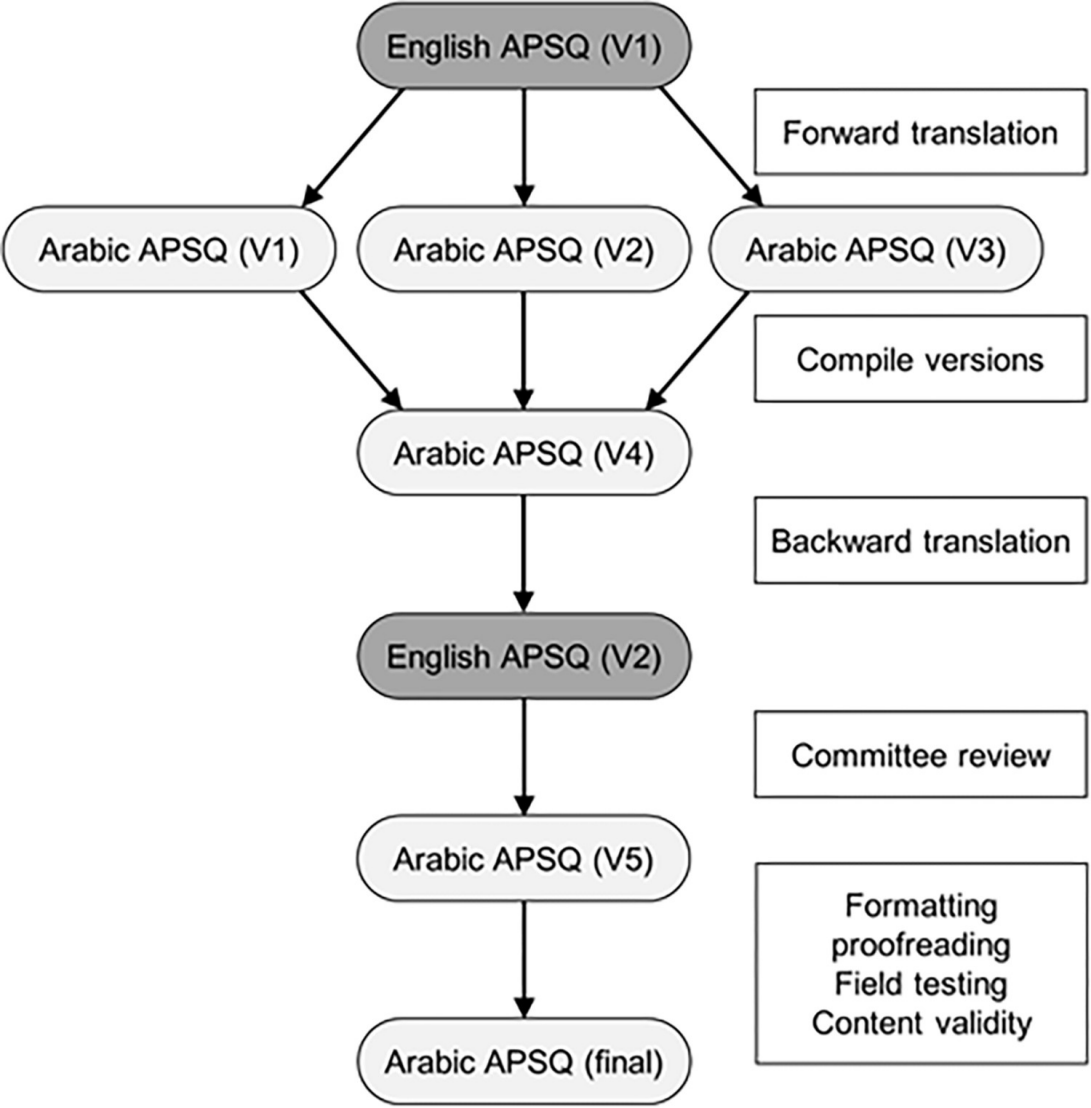


Cross-cultural adaptation measures using a forward-backward translation method were used to translate the English APSQ into the Arabic version in a manner which preserves content validity [19]. The process is outlined in Fig 1. Three independent Arabic language translations (V1-V3) were created by individuals fluent in both Arabic and English. A synthesis of the three initial translations was then produced (V4). The synthesis was then back translated into English by an individual who was blind to the original version. This version was then compared to the original English version by committee review, and any discrepancies were resolved. The synthesized Arabic version was evaluated qualitatively for semantic, idiomatic, experiential, and conceptual equivalence, leading to a pre-final version (V5). After formatting and proofreading, this version was field tested for content validity, and a final version of the Arabic APSQ was produced, which was distributed to participants electronically.

### 2.3. Statistical analysis

Statistical analysis was performed with IBM SPSS Statistics 25 (IBM, Armonk, New York, US). For statistical analysis, the 5-point Likert scale was transformed into a visual analogue scale (VAS) between 0 and 10. The items of the APSQ were statistically analysed within the classical test theory model to evaluate the psychometric characteristics of the remaining items [20, 21]. A *p*-value <0.05 was considered statistically significant. The Kolmogorov-Smirnov test and graphical examination were used to assess the data distribution. Items that were either not answered or answered as ‘not applicable’ were treated as missing values. The maximum number of incomplete answers for the validation analyses was set at three items per subject; if this number was exceeded, then the subject was excluded.

### 2.4. Item analysis

The discrimination index and homogeneity of items were examined in order to determine the effects of individual questionnaire items on the total score.

The discrimination index indicates the extent to which performance on an individual item correlates with the total score. A high correlation suggests that the item has a larger impact on the total score. Items with a discrimination index of 0.40 or higher were retained; 0.30–0.39 could possibly be improved; 0.20 to 0.29 were considered marginal and needs to be revised; and items below 0.19 were considered poor and needed to be majorly revised or discarded [22].

Item homogeneity measures the extent to which individual items correlate with the total score, and thereby reflect the extent to which the questionnaire items are measuring the same underlying construct (in this case, user satisfaction) [23].

### 2.5 Scale analysis

#### 2.5.1. Reliability

Internal consistency was tested using Cronbach’s α. Guttman split-half-coefficient was calculated to estimate the full test reliability of the questionnaire based on split-half measures, whereby the data was split into odd and even numbered items. Typically, an α of 0.7 or above is considered an acceptable level for internal consistency [24, 25].

#### 2.5.2. Construct validity

To check the underlying factor structure of the items, exploratory factor analysis was used with a rotated quartimax factor solution, with principal component analysis as the extraction method [26]. To test the suitability of the items for factor analysis, the KMO test [27] and the Bartlett test of sphericity [28] were performed as measures of sampling adequacy. Factor loadings with absolute values greater than 0.40 were considered significant and assigned to the appropriate factor.

### 2.6 Influence of additional factors on user satisfaction

Additional analyses were performed to investigate the impact of different variables on user satisfaction as depicted by the total score, calculated by averaging the VAS rating for all 15 items. Age was correlated with the APSQ total score applying the Pearson correlation. The influence of gender on user satisfaction was examined using the Mann-Whitney U-test. Univariate ANOVA was applied to test the effect of usage time of the hearing system.

## 3. Results

### 3.1. User satisfaction

**Fig 2:**
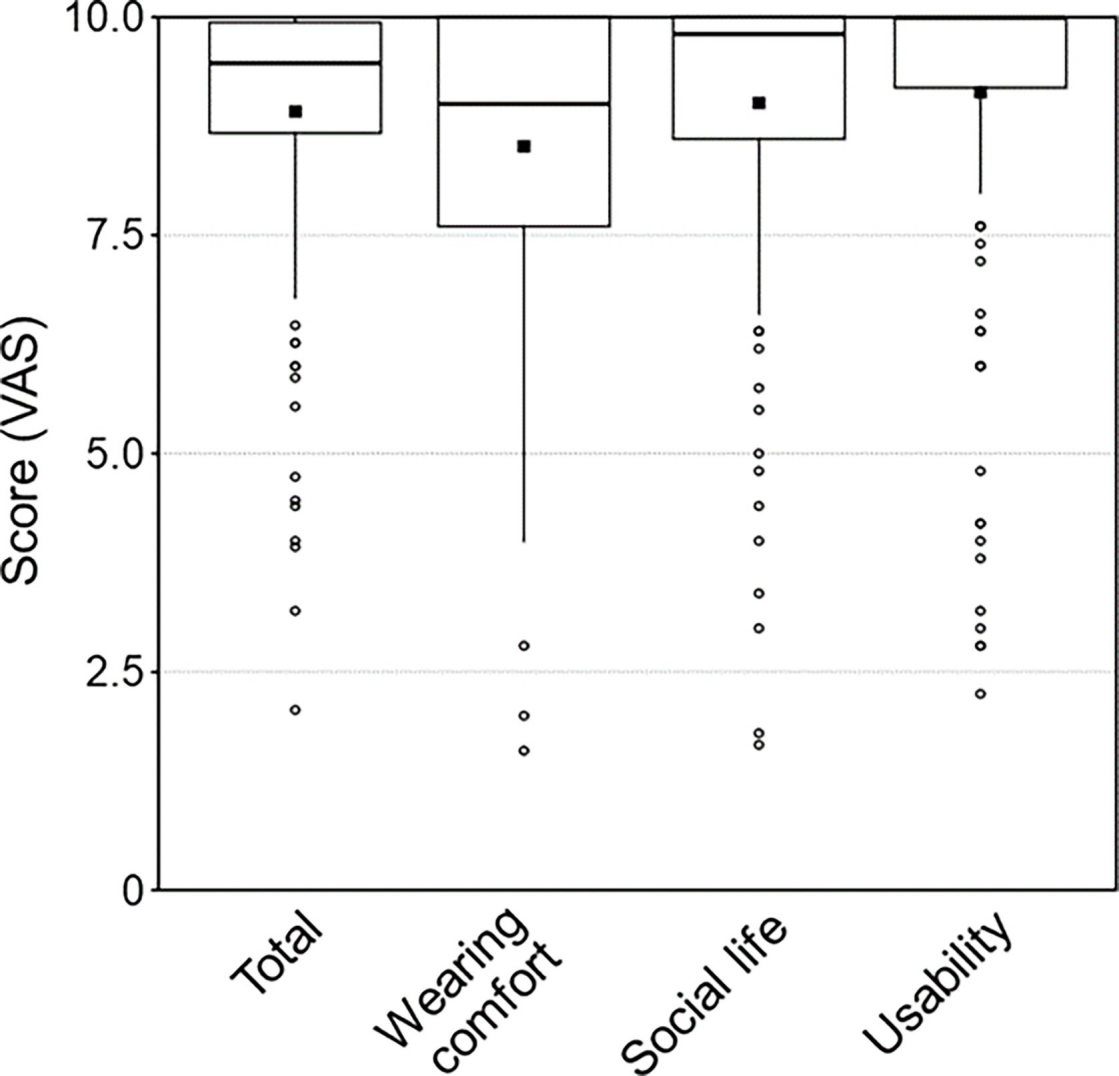


Satisfaction scores for the total scale and subscales are depicted in Fig 2. The mean total score for all participants was 8.9 (SD ± 1.5). The mean scores for the subscales were *wearing comfort*: 8.5 (SD ± 1.6), *social life*: 9.0 (SD ± 1.6), and *usability*: 9.1 (SD ± 1.7).

Item 5 (*it is easy to change the batteries of my audio processor*) had the highest number of ratings of 10 (146). Item 9 (*I can comfortably wear glasses and my audio processor at the same time*) had the highest number of ratings of 0 (13).

The mean scores per item ranged from 7.6 to 9.4 (Table 2). The highest mean score was for item 7 (*wearing the audio processor helps me live a more independent life*), while the lowest mean score was for item 9. This was also the item with the highest variance. A tendency towards a ceiling effect was observed with individual items, subscale scores, and total scores.

**Table 2:** Analysis of individual APSQ items. Asterisks indicate *p*-value < 0.001.

Subscale	Item	Mean (±SD)	Variance	Discrimination index	Pearson’s r	Factor loading (C1)
Comfort	3	8.66 (2.17)	4.72	0.64	0.70*	0.70
	6	8.91 (1.91)	3.65	0.71	0.76*	0.76
	9	7.61 (3.28)	10.79	0.46	0.57*	0.47
	12	8.86 (1.97)	3.88	0.55	0.61*	0.59
	15	8.50 (2.35)	5.51	0.58	0.66*	0.58
Social Life	1	9.40 (1.64)	2.68	0.73	0.67*	0.82
	4	8.24 (2.54)	6.45	0.65	0.72*	0.68
	7	9.46 (1.53)	2.35	0.65	0.68*	0.74
	10	9.20 (1.98)	3.94	0.72	0.73*	0.76
	13	9.11 (1.83)	3.35	0.74	0.78*	0.79
Usability	2	9.04 (2.28)	5.18	0.72	0.78*	0.77
* *	5	9.35 (1.92)	3.69	0.65	0.72*	0.75
* *	8	9.40 (1.79)	3.19	0.66	0.73*	0.74
* *	11	8.78 (2.42)	5.84	0.64	0.70*	0.72
* *	14	9.20 (1.97)	3.87	0.65	0.73*	0.70

Not all subjects answered all items, in part because some items were not individually applicable, such as item 9. Eight subjects were excluded from analysis due to non-completion of more than three items.

### 3.2 Item homogeneity

All items showed a significant Pearson’s *r* correlation between individual score and total score (*p* < 0.001), showing good item homogeneity (Table 2). The lowest correlation with total score was observed with item 9 (*I can comfortably wear glasses and my audio processor at the same time*) (0.57) and the highest was with item 2 (*It is easy to put the audio processor back on its proper place on my head*) (0.78). All items showed a discrimination index above 0.4 (Table 2).

### 3.3 Questionnaire reliability and construct validity

The questionnaire had high internal consistency and good reliability (Cronbach’s α = 0.918; Guttman’s split-half coefficient = 0.900).

The KMO test of sampling adequacy yielded a value of 0.89, indicating suitability for factor analysis. The Bartlett test of sphericity indicated a significant correlation between the items (χ^2^ = 1303.037, df = 105, *p* < 0.001). Accordingly, a factor analysis using the quartimax method was carried out. The items loaded onto one factor (C1) and explained 58.5% of the total variance, indicating that the items depict a similar construct (Table 2).

### 3.4. Influence of additional factors on user satisfaction

No significant correlation was found between total score and subject age (*Pearson’s r* = -0.043; *p* = 0.575), sex (*Mann-Whitney U* = 3536.5; *p* = 0.726), or hearing system usage time (ANOVA: F(4; 162) = 1.427, *p* = 0.227).

### 3.5 Comparison of the Arabic to the German and English APSQ scores

**Fig 3:**
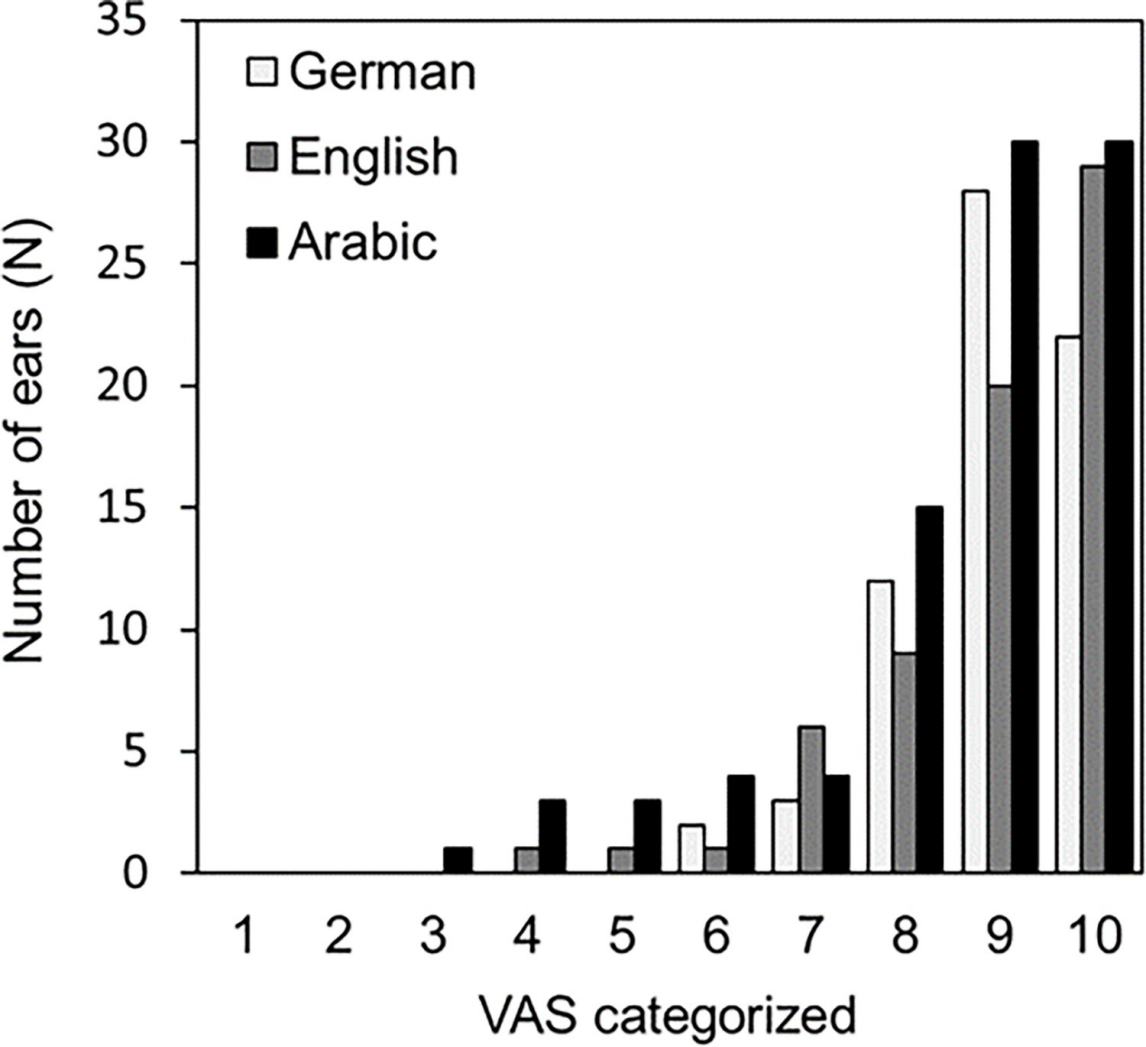


The distribution of total scores for the Arabic APSQ cohort was compared with those previously acquired for German- and English-speaking cohorts (Fig 3). The ceiling effect seen in Fig 2 is evident, albeit somewhat less pronounced in the German and English datasets.

## 4. Discussion

In this study, we aimed to construct and validate a translation of the APSQ into the Arabic language. Cross-cultural adaptation measures were used to translate the APSQ, which was then administered to a cohort of cochlear implant users. From the data obtained, we confirmed the validity of the translated questionnaire and derived information about audio processor satisfaction among this population.

In evaluating the questionnaire, itself, we found that the individual items showed a high level of item homogeneity and correlation with the total score, indicating that this version of the instrument measures a single underlying construct–user satisfaction. The Cronbach’s α and Guttman split-half-coefficient also demonstrated good test reliability and high internal consistency.

In terms of user responses, we observed a high level of satisfaction; reflected in the total score, in the three subscales, and in individual items. Users seem on the whole to be quite satisfied with their audio processors. The item with the lowest mean score (7.61) (SD ± 3.28) was item 9 (*I can comfortably wear glasses and my audio processor at the same time*). This item also showed the highest variance in responses. The variability of responses here may be related to the different wearing sites of audio processors (behind-the-ear vs. off-the-ear), as well as the style of glasses worn; one would expect some combinations to be more difficult than others.

In comparison to the original study introducing the APSQ [11] some differences are evident. Generally higher levels of audio processor satisfaction were observed in this study, with a stronger ceiling effect. One possible cause of this is the wider array of hearing implant types evaluated in the original study, which included users of cochlear implants, electric acoustic stimulation systems, middle ear implants, and bone conduction devices. Here, only cochlear implant users were evaluated. Differences in satisfaction among users of different hearing implant types have been previously reported [29, 30].

The age of study participants also differed considerably; in this study the majority of participants were children, who were excluded from the original study. In the case of young children, the questionnaire was completed on the participant’s behalf by their parent or guardian. This may conceivably lead to some bias in the responses in these cases.

## 5. Conclusion

The Arabic version of the APSQ is a useful and accessible instrument to evaluation user satisfaction with cochlear implant audio processors. The validated Arabic APSQ may improve the reliability and robustness of communication among professionals, and we encourage its diffusion into clinical practice.

## Supporting information

S1 File The English version of the Audio Processor Satisfaction Questionnaire (APSQ). (PDF)

S2 File The Arabic version of the Audio Processor Satisfaction Questionnaire (APSQ). (PDF)

S1 Data Data gathered from patients who participated in this project. (XLSX)

## Abbreviations

AP: Audio processor
APSQ: Audio Processor Satisfaction Questionnaire
GBI: Glasgow Benefit Inventory
HISQUI19: Hearing Implant Sound Quality Index
SSQ: Speech, Spatial and Qualities of Hearing Scale
VAS: Visual Analogue Scale
