# Supplementary material for: Validation of the Arabic language version of the Audio Processor Satisfaction Questionnaire (APSQ) for hearing implant users
Source: PLoS One. 2024 Jun 10;19(6):e0303301. doi: 10.1371/journal.pone.0303301 (PMC11164331; doi:10.1371/journal.pone.0303301)
Supplement: S1 File — (PDF) [file pone.0303301.s001.pdf]

Audio Processor
Satisfaction Questionnaire (APSQ)

INSTRUCTIONS

The following questions refer to how much you like or dislike using your audio processor in everyday life.

Please rate how much you like or dislike using your audio processor on a scale between 0 and 10, with a line and not a circle or an X (see example below).

A score of 10 means that you fully agree with the sentence. A score of 0 means that you do not agree at all with the sentence.

If a sentence does not apply to you, please tick “Does not apply” and leave the scale blank.

Thank you very much for your cooperation!

EXAMPLE:

Correct:

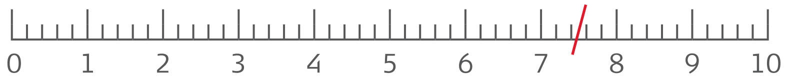

Incorrect:

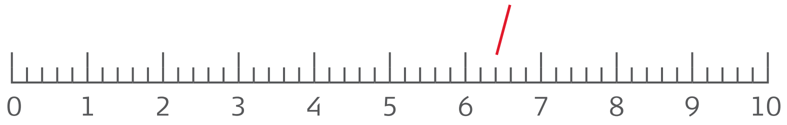

Please fill in the following information:

Today's date: _____ /_____ /_________ day/month/year

Age: ____________________ years

Gender: ☐ Female ☐ Male

LEFT EAR

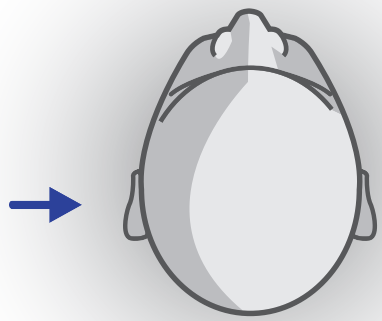

☐ Normal hearing

☐ Hearing loss

What do you wear on the LEFT side?

☐ No device

☐ A hearing aid

☐ A hearing implant system, to be specific a:

- ☐ Cochlear implant
- ☐ Cochlear implant + integrated hearing aid (EAS)
- ☐ Middle ear implant
- ☐ Bone conduction implant
- ☐ Brainstem implant

When did you receive your hearing implant?

______ /___________ month/year

What model is your current audio processor?

____

e.g. Rondo, Sonnet, Samba

How many hours a day do you wear your audio processor?

Never	0-3 h	3-6 h	6-9 h	9-12 h	>12h

RIGHT EAR

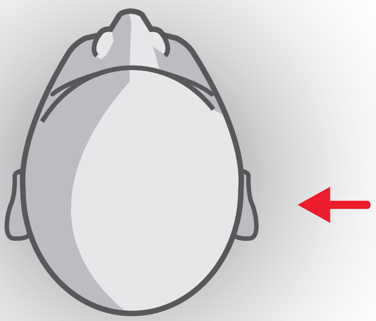

☐ Normal hearing

☐ Hearing loss

What do you wear on the RIGHT side?

☐ No device

☐ A hearing aid

☐ A hearing implant system, to be specific a:

- ☐ Cochlear implant
- ☐ Cochlear implant + integrated hearing aid (EAS)
- ☐ Middle ear implant
- ☐ Bone conduction implant
- ☐ Brainstem implant

When did you receive your hearing implant?

______ /___________ month/year

What model is your current audio processor?

____

e.g. Rondo, Sonnet, Samba

How many hours a day do you wear your audio processor?

Never	0-3 h	3-6 h	6-9 h	9-12 h	>12h

LEFT EAR

1. I feel safer and more confident when I wear my audio processor.		☐ does not apply
I do not agree at all	0 1 2 3 4 5 6 7 8 9 10	I fully agree

2. It is easy to put the audio processor on its proper place on my head.		☐ does not apply
I do not agree at all	0 1 2 3 4 5 6 7 8 9 10	I fully agree

3. My audio processor is skin-friendly (no sweating, redness, itching, etc.).		☐ does not apply
I do not agree at all	0 1 2 3 4 5 6 7 8 9 10	I fully agree

4. My audio processor allows me to have a physically active lifestyle.		☐ does not apply
I do not agree at all	0 1 2 3 4 5 6 7 8 9 10	I fully agree

5. It is easy to change the batteries of my audio processor.		☐ does not apply
I do not agree at all	0 1 2 3 4 5 6 7 8 9 10	I fully agree

6. My audio processor is comfortable to wear (no uncomfortable pressure, no feeling that the processor is heavy or bulky).		☐ does not apply
I do not agree at all	0 1 2 3 4 5 6 7 8 9 10	I fully agree

7. Wearing the audio processor helps me live a more independent life.		☐ does not apply
I do not agree at all	0 1 2 3 4 5 6 7 8 9 10	I fully agree

8. It is easy to switch the processor ON and OFF.		☐ does not apply
I do not agree at all	0 1 2 3 4 5 6 7 8 9 10	I fully agree

LEFT EAR

9. I can comfortably wear glasses and my audio processor at the same time.	☐ does not apply
I do not agree at all — 0 1 2 3 4 5 6 7 8 9 10	I fully agree

10. My audio processor makes it easier for me to enjoy cultural activities (e.g. theatre, cinema).	☐ does not apply
I do not agree at all — 0 1 2 3 4 5 6 7 8 9 10	I fully agree

11. My audio processor functions properly (does not switch off for no reason).	☐ does not apply
I do not agree at all — 0 1 2 3 4 5 6 7 8 9 10	I fully agree

12. I can comfortably wear head-wear (e.g. hat, helmet) and my audio processor at the same time.	☐ does not apply
I do not agree at all — 0 1 2 3 4 5 6 7 8 9 10	I fully agree

13. My audio processor makes it easier for me to enjoy social activities (e.g. joining conversations, meeting new people, going out).	☐ does not apply
I do not agree at all — 0 1 2 3 4 5 6 7 8 9 10	I fully agree

14. My audio processor is easy to take care of (e.g. to clean, to dry).	☐ does not apply
I do not agree at all — 0 1 2 3 4 5 6 7 8 9 10	I fully agree

15. My audio processor stays in the same position all day.	☐ does not apply
I do not agree at all — 0 1 2 3 4 5 6 7 8 9 10	I fully agree

RIGHT EAR

1. I feel safer and more confident when I wear my audio processor.	☐ does not apply
I do not agree at all — 0 1 2 3 4 5 6 7 8 9 10	I fully agree

2. It is easy to put the audio processor on its proper place on my head.	☐ does not apply
I do not agree at all — 0 1 2 3 4 5 6 7 8 9 10	I fully agree

3. My audio processor is skin-friendly (no sweating, redness, itching, etc.).	☐ does not apply
I do not agree at all — 0 1 2 3 4 5 6 7 8 9 10	I fully agree

4. My audio processor allows me to have a physically active lifestyle.	☐ does not apply
I do not agree at all — 0 1 2 3 4 5 6 7 8 9 10	I fully agree

5. It is easy to change the batteries of my audio processor.	☐ does not apply
I do not agree at all — 0 1 2 3 4 5 6 7 8 9 10	I fully agree

6. My audio processor is comfortable to wear (no uncomfortable pressure, no feeling that the processor is heavy or bulky).	☐ does not apply
I do not agree at all — 0 1 2 3 4 5 6 7 8 9 10	I fully agree

7. Wearing the audio processor helps me live a more independent life.	☐ does not apply
I do not agree at all — 0 1 2 3 4 5 6 7 8 9 10	I fully agree

8. It is easy to switch the processor ON and OFF.	☐ does not apply
I do not agree at all — 0 1 2 3 4 5 6 7 8 9 10	I fully agree

RIGHT EAR

9. I can comfortably wear glasses and my audio processor at the same time.	☐ does not apply
I do not agree at all — 0 1 2 3 4 5 6 7 8 9 10	I fully agree

10. My audio processor makes it easier for me to enjoy cultural activities (e.g. theatre, cinema).	☐ does not apply
I do not agree at all — 0 1 2 3 4 5 6 7 8 9 10	I fully agree

11. My audio processor functions properly (does not switch off for no reason).	☐ does not apply
I do not agree at all — 0 1 2 3 4 5 6 7 8 9 10	I fully agree

12. I can comfortably wear head-wear (e.g. hat, helmet) and my audio processor at the same time.	☐ does not apply
I do not agree at all — 0 1 2 3 4 5 6 7 8 9 10	I fully agree

13. My audio processor makes it easier for me to enjoy social activities (e.g. joining conversations, meeting new people, going out).	☐ does not apply
I do not agree at all — 0 1 2 3 4 5 6 7 8 9 10	I fully agree

14. My audio processor is easy to take care of (e.g. to clean, to dry).	☐ does not apply
I do not agree at all — 0 1 2 3 4 5 6 7 8 9 10	I fully agree

15. My audio processor stays in the same position all day.	☐ does not apply
I do not agree at all — 0 1 2 3 4 5 6 7 8 9 10	I fully agree
