# Supplementary material for: Validation of the Arabic language version of the Audio Processor Satisfaction Questionnaire (APSQ) for hearing implant users
Source: PLoS One. 2024 Jun 10;19(6):e0303301. doi: 10.1371/journal.pone.0303301 (PMC11164331; doi:10.1371/journal.pone.0303301)
Supplement: S2 File — (PDF) [file pone.0303301.s002.pdf]

# استبيان لقياس مدى الرضا عن معالج الصوت (APSQ)

## تعليمات

تشير الأسئلة التالية إلى مدى رضاك أو عدم رضاك عن استخدام معالج الصوت الخاص بك في الحياة اليومية.

يرجى تقييم مدى رضاك أو عدم رضاك بمقياس يتراوح بين ٠ و١٠، وذلك بوضع خط مائل وليس بوضع دائرة أو علامة X (انظر المثال أدناه).

الدرجة (١٠) تعني أنك تتفق تمامًا مع الجملة. ودرجة (٠) تعني أنك لا تتفق مع الجملة على الإطلاق.

إذا كانت الجملة لا تنطبق عليك، يرجى وضع علامة على "لا تنطبق" وترك المقياس فارغاً.

شكراً جزيلاً،

## مثال

صحيح

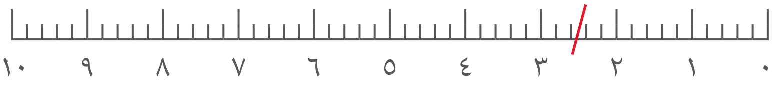


غير صحيح

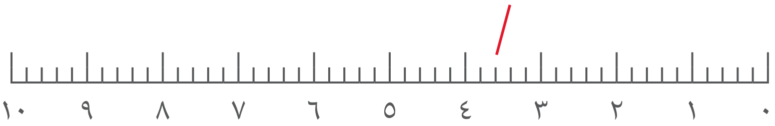


## الرجاء تعبئة البيانات التالية

تاريخ اليوم: ________ / ________ / ________ (يوم / شهر / سنة)

العمر: ________ سنة

الجنس: ☐ ذكر ☐ أنثى

## الأذن اليمنى

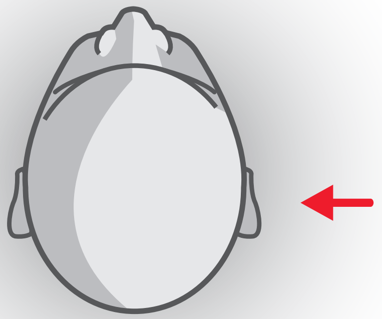

☐ سمع طبيعي
☐ يوجد ضعف سمع

ماذا تلبس في الأذن اليمنى؟

☐ لا شيء
☐ سماعة
☐ زرعة، وبشكل محدد
  - ☐ زرعة قوقعة
  - ☐ زرعة قوقعة مع سماعة مدمجة
  - ☐ زرعة أذن وسطى
  - ☐ زرعة توصيل عظمي
  - ☐ زرعة جذع دماغ

متى حصلت على زرعة الأذن؟

__________/ ______ (الشهر/السنة)

ما هو نوع السماعة الخارجية التي تستخدمها؟

____________________

(على سبيل المثال: أوبس ٢، سونت، ساميا)

كم عدد الساعات التي تستخدم فيها معالج الصوت بشكل يومي؟

| لا أستخدمها | ٠-٣ ساعات | ٣-٦ ساعات | ٦-٩ ساعات | ٩-١٢ ساعة | أكثر من ١٢ ساعة |
|---|---|---|---|---|---|
| | | | | | |

## الأذن اليسرى

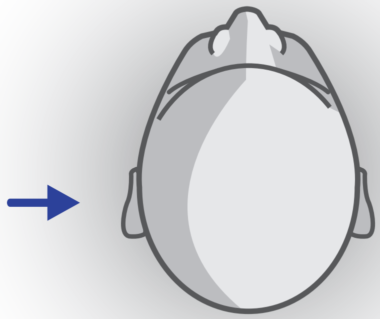

☐ سمع طبيعي
☐ يوجد ضعف سمع

ماذا تلبس في الأذن اليمنى؟

☐ لا شيء
☐ سماعة
☐ زرعة، وبشكل محدد
  - ☐ زرعة قوقعة
  - ☐ زرعة قوقعة مع سماعة مدمجة
  - ☐ زرعة أذن وسطى
  - ☐ زرعة توصيل عظمي
  - ☐ زرعة جذع دماغ

متى حصلت على زرعة الأذن؟

__________/ ______ (الشهر/السنة)

ما هو نوع السماعة الخارجية التي تستخدمها؟

____________________

(على سبيل المثال: أوبس ٢، سونت، ساميا)

كم عدد الساعات التي تستخدم فيها معالج الصوت بشكل يومي؟

| لا أستخدمها | ٠-٣ ساعات | ٣-٦ ساعات | ٦-٩ ساعات | ٩-١٢ ساعة | أكثر من ١٢ ساعة |
|---|---|---|---|---|---|
| | | | | | |

## الأذن اليسرى

| | | |
|---|---|---|
| ١. | أشعر بأمان وثقة أكثر عند لبس معالج الصوت. | ☐ لا ينطبق |
| لا أتفق على الإطلاق | ٠ ١ ٢ ٣ ٤ ٥ ٦ ٧ ٨ ٩ ١٠ | أتفق تماماً |
| ٢. | يمكنني وضع معالج الصوت بسهولة في المكان المناسب على رأسي. | ☐ لا ينطبق |
| لا أتفق على الإطلاق | ٠ ١ ٢ ٣ ٤ ٥ ٦ ٧ ٨ ٩ ١٠ | أتفق تماماً |
| ٣. | معالج الصوت مريح لبشرتي (لا يسبب عرق أو حكة أو احمرار أو ما إلى ذلك). | ☐ لا ينطبق |
| لا أتفق على الإطلاق | ٠ ١ ٢ ٣ ٤ ٥ ٦ ٧ ٨ ٩ ١٠ | أتفق تماماً |
| ٤. | معالج الصوت يسمح لي بنمط حياة نشط بدنياً (مثل القيام بأنشطة الرياضة). | ☐ لا ينطبق |
| لا أتفق على الإطلاق | ٠ ١ ٢ ٣ ٤ ٥ ٦ ٧ ٨ ٩ ١٠ | أتفق تماماً |
| ٥. | يمكنني بسهولة تغيير البطارية الخاصة بمعالج الصوت. | ☐ لا ينطبق |
| لا أتفق على الإطلاق | ٠ ١ ٢ ٣ ٤ ٥ ٦ ٧ ٨ ٩ ١٠ | أتفق تماماً |
| ٦. | معالج الصوت الخاص بي مريح في اللبس (لا يسبب ضغط ولا يوجد شعور بأنه ثقيل أو ضخم). | ☐ لا ينطبق |
| لا أتفق على الإطلاق | ٠ ١ ٢ ٣ ٤ ٥ ٦ ٧ ٨ ٩ ١٠ | أتفق تماماً |
| ٧. | لبس معالج الصوت يساعدني على العيش باستقلالية أكثر وعدم الحاجة للأخرين. | ☐ لا ينطبق |
| لا أتفق على الإطلاق | ٠ ١ ٢ ٣ ٤ ٥ ٦ ٧ ٨ ٩ ١٠ | أتفق تماماً |
| ٨. | أستطيع تشغيل وإطفاء معالج الصوت بسهولة. | ☐ لا ينطبق |
| لا أتفق على الإطلاق | ٠ ١ ٢ ٣ ٤ ٥ ٦ ٧ ٨ ٩ ١٠ | أتفق تماماً |

## الأذن اليسرى

| | | |
|---|---|---|
| ٩. | أستطيع لبس النظارة ومعالج الصوت الخاص بي بشكل مريح في نفس الوقت. | ☐ لا ينطبق |
| لا أتفق على الإطلاق | ٠ ١ ٢ ٣ ٤ ٥ ٦ ٧ ٨ ٩ ١٠ | أتفق تماماً |

| | | |
|---|---|---|
| ١٠. | لبس معالج الصوت يسهل الاستمتاع بالأنشطة الثقافية (مثل السينما والمسرح). | ☐ لا ينطبق |
| لا أتفق على الإطلاق | ٠ ١ ٢ ٣ ٤ ٥ ٦ ٧ ٨ ٩ ١٠ | أتفق تماماً |

| | | |
|---|---|---|
| ١١. | معالج الصوت الخاص بي يعمل بشكل جيد (لا يتوقف عن العمل دون سبب). | ☐ لا ينطبق |
| لا أتفق على الإطلاق | ٠ ١ ٢ ٣ ٤ ٥ ٦ ٧ ٨ ٩ ١٠ | أتفق تماماً |

| | | |
|---|---|---|
| ١٢. | أستطيع ارتداء أغطية الرأس (مثل الطاقية، الشماغ، الحجاب، الخوذة) مع معالج الصوت في نفس الوقت بسهولة. | ☐ لا ينطبق |
| لا أتفق على الإطلاق | ٠ ١ ٢ ٣ ٤ ٥ ٦ ٧ ٨ ٩ ١٠ | أتفق تماماً |

| | | |
|---|---|---|
| ١٣. | معالج الصوت يسهل الاستمتاع بالأنشطة الاجتماعية (مثل الدخول في محادثات، الالتقاء بأشخاص جدد، والخروج). | ☐ لا ينطبق |
| لا أتفق على الإطلاق | ٠ ١ ٢ ٣ ٤ ٥ ٦ ٧ ٨ ٩ ١٠ | أتفق تماماً |

| | | |
|---|---|---|
| ١٤. | من السهل العناية بمعالج الصوت الخاص بي (مثل التنظيف والتجفيف). | ☐ لا ينطبق |
| لا أتفق على الإطلاق | ٠ ١ ٢ ٣ ٤ ٥ ٦ ٧ ٨ ٩ ١٠ | أتفق تماماً |

| | | |
|---|---|---|
| ١٥. | معالج الصوت الخاص بي يبقى ثابتاً في مكانه طوال اليوم (لا يسقط). | ☐ لا ينطبق |
| لا أتفق على الإطلاق | ٠ ١ ٢ ٣ ٤ ٥ ٦ ٧ ٨ ٩ ١٠ | أتفق تماماً |

## الأذن اليمنى

| | | |
|---|---|---|
| ١. | أشعر بأمان وثقة أكثر عند لبس معالج الصوت. | ☐ لا ينطبق |
| لا أتفق على الإطلاق | ٠ ١ ٢ ٣ ٤ ٥ ٦ ٧ ٨ ٩ ١٠ | أتفق تماماً |
| ٢. | يمكنني وضع معالج الصوت بسهولة في المكان المناسب على رأسي. | ☐ لا ينطبق |
| لا أتفق على الإطلاق | ٠ ١ ٢ ٣ ٤ ٥ ٦ ٧ ٨ ٩ ١٠ | أتفق تماماً |
| ٣. | معالج الصوت مريح لبشرتي (لا يسبب عرق أو حكة أو احمرار أو ما إلى ذلك). | ☐ لا ينطبق |
| لا أتفق على الإطلاق | ٠ ١ ٢ ٣ ٤ ٥ ٦ ٧ ٨ ٩ ١٠ | أتفق تماماً |
| ٤. | معالج الصوت يسمح لي بنمط حياة نشط بدنياً (مثل القيام بأنشطة الرياضة). | ☐ لا ينطبق |
| لا أتفق على الإطلاق | ٠ ١ ٢ ٣ ٤ ٥ ٦ ٧ ٨ ٩ ١٠ | أتفق تماماً |
| ٥. | يمكنني بسهولة تغيير البطارية الخاصة بمعالج الصوت. | ☐ لا ينطبق |
| لا أتفق على الإطلاق | ٠ ١ ٢ ٣ ٤ ٥ ٦ ٧ ٨ ٩ ١٠ | أتفق تماماً |
| ٦. | معالج الصوت الخاص بي مريح في اللبس (لا يسبب ضغط ولا يوجد شعور بأنه ثقيل أو ضخم). | ☐ لا ينطبق |
| لا أتفق على الإطلاق | ٠ ١ ٢ ٣ ٤ ٥ ٦ ٧ ٨ ٩ ١٠ | أتفق تماماً |
| ٧. | لبس معالج الصوت يساعدني على العيش باستقلالية أكثر وعدم الحاجة للأخرين. | ☐ لا ينطبق |
| لا أتفق على الإطلاق | ٠ ١ ٢ ٣ ٤ ٥ ٦ ٧ ٨ ٩ ١٠ | أتفق تماماً |
| ٨. | أستطيع تشغيل وإطفاء معالج الصوت بسهولة. | ☐ لا ينطبق |
| لا أتفق على الإطلاق | ٠ ١ ٢ ٣ ٤ ٥ ٦ ٧ ٨ ٩ ١٠ | أتفق تماماً |

## الأذن اليمنى

| | | |
|---|---|---|
| .٩ | أستطيع لبس النظارة ومعالج الصوت الخاص بي بشكل مريح في نفس الوقت. | ☐ لا ينطبق |
| لا أتفق على الإطلاق | ٠ ١ ٢ ٣ ٤ ٥ ٦ ٧ ٨ ٩ ١٠ | أتفق تماماً |

| | | |
|---|---|---|
| .١٠ | لبس معالج الصوت يسهل الاستمتاع بالأنشطة الثقافية (مثل السينما والمسرح). | ☐ لا ينطبق |
| لا أتفق على الإطلاق | ٠ ١ ٢ ٣ ٤ ٥ ٦ ٧ ٨ ٩ ١٠ | أتفق تماماً |

| | | |
|---|---|---|
| .١١ | معالج الصوت الخاص بي يعمل بشكل جيد (لا يتوقف عن العمل دون سبب). | ☐ لا ينطبق |
| لا أتفق على الإطلاق | ٠ ١ ٢ ٣ ٤ ٥ ٦ ٧ ٨ ٩ ١٠ | أتفق تماماً |

| | | |
|---|---|---|
| .١٢ | أستطيع ارتداء أغطية الرأس (مثل الطاقية، الشماغ، الحجاب، الخوذة) مع معالج الصوت في نفس الوقت بسهولة. | ☐ لا ينطبق |
| لا أتفق على الإطلاق | ٠ ١ ٢ ٣ ٤ ٥ ٦ ٧ ٨ ٩ ١٠ | أتفق تماماً |

| | | |
|---|---|---|
| .١٣ | معالج الصوت يسهل الاستمتاع بالأنشطة الاجتماعية (مثل الدخول في محادثات، الالتقاء بأشخاص جدد، والخروج). | ☐ لا ينطبق |
| لا أتفق على الإطلاق | ٠ ١ ٢ ٣ ٤ ٥ ٦ ٧ ٨ ٩ ١٠ | أتفق تماماً |

| | | |
|---|---|---|
| .١٤ | من السهل العناية بمعالج الصوت الخاص بي (مثل التنظيف والتجفيف). | ☐ لا ينطبق |
| لا أتفق على الإطلاق | ٠ ١ ٢ ٣ ٤ ٥ ٦ ٧ ٨ ٩ ١٠ | أتفق تماماً |

| | | |
|---|---|---|
| .١٥ | معالج الصوت الخاص بي يبقى ثابتاً في مكانه طوال اليوم (لا يسقط). | ☐ لا ينطبق |
| لا أتفق على الإطلاق | ٠ ١ ٢ ٣ ٤ ٥ ٦ ٧ ٨ ٩ ١٠ | أتفق تماماً |
